# The complete chloroplast genome of *Choerospondias axillaris* (Roxb.) B. L. Burtt et A. W. Hill, an ancient and versatile plant

**DOI:** 10.1080/23802359.2021.1952120

**Published:** 2021-07-15

**Authors:** Kun Zhang, Xun Zhang, Qi Wang, Xiaofei Shan

**Affiliations:** College of Life Sciences, Shanxi Datong University, Datong, PR China

**Keywords:** *Choerospondias axillaris*, Anacardiaceae, chloroplast genome, Illumina sequencing

## Abstract

*Choerospondias axillaris*, an ancient and versatile plant of Anacardiaceae, which is widespread in eastern Asia. The complete chloroplast (cp) genome of *C. axillaris* was generated and determined in this work. The genome of cp was 159,131 bp in length with a total GC content of 37.6%. The circular molecular genome presented a quadripartite structure, comprising a 91,305 bp large single-copy region (LSC), a 19,092 bp small single-copy region (SSC), and a pair of inverted repeat regions with 24,367 bp. The complete genome encoded 128 genes, including 83 protein coding, 37 *tRNA* and eight *rRNA* genes. Phylogenetic analysis indicated that *C. axillaris* was most related to *Sclerocarya birrea.*

*Choerospondias axillaris* (Roxb.) B. L. Burtt et A. W. Hill is a deciduous broad-leaved tree species of Anacardiaceae, which is a monospecific species of genus *Choerospondias* in eastern Asia and mainly distributed in Japan and South China. *C. axillaris* has a long cultivation and utilization history, whose fruit fossil with 15-million-year-old was first discovered in Fujian, China (Wang et al. 2020). For its characteristics of fast growth and strong adaptability, *C. axillaris* is suitable for artificial afforestation. So that *C. axillaris* has a growing popularity and important economic effects, it is noted for timber, edible, and medicinal values. Previous researches on *C. axillaris* have mainly focused on pharmacological action (Sun et al. 2015; Mann et al. 2020), phytochemical investigation (Li et al. 2018), photosynthetic physiology (Li and Xu 2020), and morphological diversity (Wang et al. 2019). However, there is no report on the chloroplast (cp) genome of *C. axillaris*. The complete cp genome has been progressively analyzed in diverse species to provide insight into species identification and population dynamics (Shaw et al. 2014). In this study, we established and characterized the complete *C. axillaris* cp genome and performed phylogenetic analysis with the cp genomes of other species of Anacardiaceae.

The leaves of *C. axillaris* were sampled from Chenshan Botanical Garden, Shanghai, China (31°08′N, 121°18′E). And the voucher specimen (Accession number: CSSH202104) was stored in Shanxi Datong University (http://www.sxdtdx.edu.cn/, Kun Zhang, 876828320@qq.com). Genomic DNA extraction of *C. axillaris* was processed according to the modified CTAB method (Murray and Thompson 1980). After DNA purification, the libraries with an average length of 350 bp were constructed using the NexteraXT DNA Library Preparation Kit (Illumina, San Diego, CA), and high-throughput sequencing was carried out on Illumina Novaseq 6000 platform. In total, 3.7 Gb clean reads were generated by editing raw sequence reads with NGS QC Tool kit (Patel and Jain 2012). The cp genome was *de novo* assembled by SPAdes version 3.11.0 software (Bankevich et al. 2012), and cp genes of *C. axillaris* were annotated by using PGA (Qu et al. 2019) through aligning to genome of *Sclerocarya birrea* (A. Rich.) Hochst cp (Accession number: NC043919). The annotated sequence was submitted to GenBank and data were openly available at (https://www.ncbi.nlm.nih.gov/nuccore/MZ042936.1/) under the accession MZ042936. The associated SRA number is SRR14328382.

The cp genome of *C. axillaris* was a circular molecular genome presented a quadripartite structure. The genome was 159,131 bp in length, and consisted of a 91,305 bp large single-copy (LSC) region, a 19,092 bp small single-copy (SSC) region, and a pair of inverted repeat (IRa and IRb) regions with 24,367 bp. The overall GC content detected in the *C. axillaris* cp genome was 37.6%. The assembled genome encoded 128 genes, including 83 protein coding, 37 *tRNA* and eight *rRNA* genes. The majority of these genes did not contain intron, while 15 genes (*atp*F, *ndh*A, *ndh*B, *pet*B, *pet*D, *rpl*2, *rpl*16, *rpo*C1, *rps*16, *trn*A-UGC, *trn*G-UCC, *trn*I-GAU, *trn*K-UUU, *trn*L-UAA, and *trn*V-UAC) contained one intron and two genes (*clp*P and *ycf*3) contained double introns. All genes occurred as a single copy, except that 10 genes (*ycf*1, *rrn*4.5, *rrn*5, *rrn*16, *rrn*23, tRNA-Ile, tRNA-Ala, tRNA-Arg, tRNA-Asn, and ORF302) were duplicated in IR regions.

To further determine the phylogenetic position of *C. axillaris*, 20 complete cp genomes of different species within Anacardiaceae family and other three species as outgroup were selected to construct a phylogenetic tree. The sequences were downloaded from NCBI GenBank database and aligned by using MAFFT (Katoh and Standley 2013). Subsequently, a maximum-likelihood phylogenetic tree was established by IQTREE version 1.6 (Jana et al. 2016) with 1000 bootstrap replicates. The main genera displayed on the phylogenetic tree were *Rhus*, *Mangifera*, *Toxicodendron*, *Pistacia*, and *Spondias*. Phylogenetic analysis revealed that *C. axillaris* exhibited the closest relationship with *Sclerocarya birrea* (Figure 1). The information derived from this work provides a basis for future genetic and evolutionary studies in *C. axillaris*, which may help facilitate the utilization and protection of this ancient and versatile plant.

**Figure 1.**
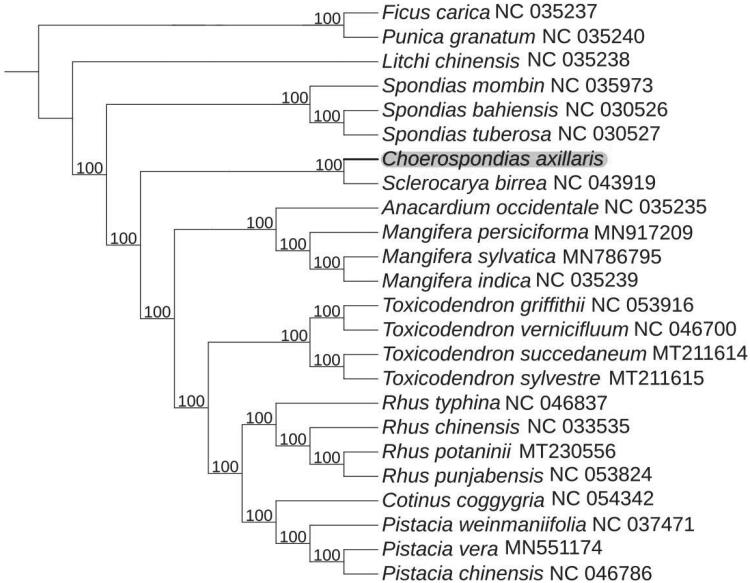
Maximum-likelihood phylogenetic tree for *C. axillaris* based on 21 complete cp genome in Anacardiaceae, with *Litchi chinensis* Sonn., *Punica granatum* L., and *Ficus carica* L. as outgroup. The bootstrap values are located on each node and the Genbank accession numbers are shown beside the Latin name of the species.

## Data Availability

The assembled complete cp genome sequence of *C. axillaris* has been submitted to GenBank of NCBI and is openly available under the accession number: MZ042936 (https://www.ncbi.nlm.nih.gov/nuccore/MZ042936.1/). The associated BioProject, SRA, and Bio-Sample numbers are PRJNA725317, SRR14328382, and SAMN18875962, respectively.
